# Neuropeptide Y receptor 1 and galanin receptor 2 (NPY1R-GALR2) interactions in the dentate gyrus and their relevance for neurogenesis and cognition

**DOI:** 10.3389/fncel.2024.1323986

**Published:** 2024-02-14

**Authors:** Rasiel Beltran-Casanueva, Aracelis Hernández-García, Paula de Amo García, Encarnación Blanco-Reina, Pedro Serrano-Castro, Natalia García-Casares, Kjell Fuxe, Dasiel O. Borroto-Escuela, Manuel Narváez

**Affiliations:** ^1^Department of Neuroscience, Karolinska Institutet, Stockholm, Sweden; ^2^Receptomics and Brain Disorders Lab, Facultad de Medicina, Edificio Lopez-Peñalver, Instituto de Investigación Biomédica de Málaga, Universidad de Málaga, Spain; ^3^Departamento de Docencia e Investigación, Universidad de Ciencias Médicas de Holguín, Hospital Pedíatrico Universitario Octavio de la Concepción de la Pedraja, Holguín, Cuba; ^4^Facultad de Medicina, Instituto de Investigación Biomédica de Málaga, Universidad de Málaga, Málaga, Spain; ^5^Grupo Hospitalario Vithas, Vithas Málaga, Málaga, Spain; ^6^Unit of Neurology, Instituto de Investigación Biomédica de Málaga, Hospital Regional Universitario de Málaga, Málaga, Spain

**Keywords:** neurogenic enhancement, cognitive enhancement, GALR2 agonist, NPY1R agonist, spatial memory performance, neuronal proliferation

## Abstract

**Introduction:**

This study may unveil novel insights into the interactions between neuropeptide Y receptor 1 (NPY1R) and galanin receptor 2 (GALR2), in the dentate gyrus of the dorsal hippocampus, shedding light on their role in neurogenesis and cognitive functions. Existing literature highlights the potential of these interactions in enhancing learning and memory, yet detailed mechanisms remain underexplored.

**Methods:**

Utilizing intracerebroventricular injections of GALR2 and NPY1R agonists in Sprague-Dawley male rats, we examined neurogenesis via markers PCNA and DCX, and memory consolidation through the object-in-place task over a three-week period.

**Results:**

Significant increases in NPY1R-GALR2 co-localization and neuroblast proliferation were observed, alongside enhanced memory consolidation. These findings suggest a synergistic effect of NPY1R and GALR2 activation on cognitive functions.

**Discussion:**

Our findings may foster the development of novel heterobivalent or multitargeting drugs, affecting NPY1R-GALR2 interaction, and suggest a future pharmacogical strategy for improving learning and memory found in many brain diseases. Further research is encouraged to explore these mechanisms in pathological models.

## Highlights

• This research demonstrates a sustained surge in neurogenesis within the dorsal dentate gyrus (DG) following intracerebroventricular injection of GALR2 and NPY1R agonists.
• It is noted that co-delivering the M1145 GALR2 agonist and the NPY1R agonist spurred neuroblast proliferation involving a protein in replication processes (PCNA +) and doublecortin (DCX +), a microtubule associated protein. However, quiescent neural progenitors and astrocytes remained unaffected.
• The study reveals a rise in positive PLA signals, indicating NPY1R-GALR2 co-localization. Significant enhancement in object-in-place memory consolidation was seen after a three-week treatment, hinting at a significant combined role of GalR2 and NPY1R receptors in memory consolidation involving a NPY1R-GalR2 specific interaction, It may offer a promising avenue for future treatment strategies of learning and memory.

## Introduction

In contemporary neuroscience, the exploration of brain functions and processes has led to the identification of key modulators that govern a myriad of physiological activities. Among these, Neuropeptide-Y (NPY) stands out as a 36-amino acid peptide that is profusely found throughout the brain, including regions such as the hippocampus (Vezzani et al., 1999). This peptide is implicated in the control of diverse biological and pathophysiological functions like feeding behaviors, neuroplasticity, memory, and learning (Stanley and Leibowitz, 1984; Sorensen et al., 2008). NPY has a role in spatial memory and learning and evidence indicates that it has both stimulatory and inhibitory effects on memory. These dual effects are dependent on an array of variables such as NPY receptor subtypes, dosage, neuroanatomical regions involved, temporal phase, and the nature of the memory (Redrobe et al., 2004; Van Den Pol, 2012; Gotzsche and Woldbye, 2016). Furthermore, NPY has been linked to spatial learning during hippocampal tasks, with studies revealing elevated levels of NPY mRNA in the dentate gyrus following recognition task exposure (Hadad-Ophir et al., 2014). Specifically, NPY Y1 receptors (NPY1R) have been identified as a critical target for improving neurogenesis in the dentate region and fostering spatial learning (Gotzsche and Woldbye, 2016). Such observations underline the importance of NPY and NPY1R signaling in hippocampal learning and memory processes.

In parallel, Galanin (GAL) is another prominent peptide widely distributed in both the central and peripheral nervous system. GAL is involved in numerous functions, such as energy homeostasis, reproduction, feeding, cognition, and learning, which has been well established (Lang et al., 2007, 2015). GAL influences the brain via its receptors GAL1-R, GAL2-R, and GAL3-R, each contributing to various effects. Particularly, GAL2-R is prevalent in several brain regions, such as the dentate gyrus of the hippocampus, and has been associated with growth-promoting activities in several neurons (O’Donnell et al., 1999; Burazin et al., 2000; Hohmann et al., 2003). The complexity of interactions between NPY and GAL extends beyond individual roles, with substantial research uncovering the ways they influence one another through NPY1R-GALR2 interactions. These interactions are noted in intricate brain regions, such as the amygdala, dorsal and ventral hippocampus, various hypothalamic regions, and the medial prefrontal cortex (Narvaez et al., 2015, 2016, 2018; Borroto-Escuela et al., 2021, 2022; Mirchandani-Duque et al., 2022; Diaz-Sanchez et al., 2023). Recent experimental studies have probed into these connections, exploring the ways NPY1R and GALR2 agonists stimulate proliferation in DG neuronal precursor cells *in vivo* at a 24-hour mark (Borroto-Escuela et al., 2022; Mirchandani-Duque et al., 2022). These findings could introduce new avenues in therapeutic interventions on learning and memory deficits.

Considering the anterograde effects of neurogenesis on learning and memory, the addition of new neurons is vital for encoding new information and contexts. The present research seeks to study the influence of NPY and GAL on the dorsal hippocampus 3 weeks following treatment. The chosen timeline is founded on previous research that suggests new granular neurons can functionally contribute to hippocampal function within 2−3 weeks (Schmidt-Hieber et al., 2004; Bruel-Jungerman et al., 2005; Snyder et al., 2009). Our methodological approach includes an examination of hippocampal cell proliferation effects mediated by NPY1R and GALR2 agonists through the use of proliferating cell nuclear antigen (PCNA). In addition, the co-localization of NPY1R-GALR2 with *in situ* proximity ligation assay (PLA). Lastly, the functional outcomes of NPY and GAL interactions on the dorsal hippocampus will be evaluated using the object-in-place task, a paradigm specific to spatial hippocampal memory.

We seek to present an integrated view of NPY and GAL interactions and their implications for the brain function and potential therapeutic applications. By weaving in historical research with cutting-edge developments, we hope to offer a comprehensive perspective on a complex and evolving field that has profound implications for understanding brain function and treating learning and memory.

## Materials and methods

### Animal housing and care

Sprague-Dawley male rats were obtained from CRIFFA, Barcelona, weighing between 200−250 grams and aged between 6−8 weeks. They were given unrestricted access to both food and water and maintained under a consistent 12-hour dark/light cycle. Environmental conditions were controlled for relative humidity (55−60%) and temperature (22 ± 2°C). The protocols for preclinical testing were conducted in compliance with the EU Directive 2010/63/EU and Spanish Directive (Real Decretory 53/2013) guidelines. The University of Málaga’s Local Animal Ethics, Care, and Use Committee, Spain (CEUMA 45-2022-A), approved all procedures concerning the experimental treatment and rat housing and maintenance.

### Preparation of drugs

Peptides were freshly diluted in artificial cerebrospinal fluid (aCSF), which consists of (in mM): 120 NaCl, 20 NaH2CO3, 2 KCl, 0.5 KH2PO4, 1.2 CaCl2, 1.8 MgCl2, 0.5 Na2SO4, and 5.8 D-glucose, pH 7.4. aCSF was utilized as a vehicle for control preparations. Agonists and antagonists for specific receptors, namely Galanin receptor 2 agonist (M1145), NPY1R receptor agonist [Leu31, Pro34] NPY, GALR2 Antagonist M871, were sourced from Tocris Bioscience (Bristol, UK). Supplementary Material contains an in-depth account of the intracerebroventricular (icv) administration of peptides

### Experimental design

The study animals were assorted into five distinctive experimental groups: (1) aCSF as a control, (2) M1145-treated (3 nmol), (3) NPY1R agonist-treated using NPYY1R agonist [Leu31, Pro34] NPY (3 nmol), (4) M1145 + NPY1R: a combination of both compounds, and (5) M1145 + NPY1R + M871: treatment with GAL, [Leu31, Pro34]NPY, and the GALR2 antagonist (M871, 3 nmol) (*n* = 4 per group). These peptides were administered once daily for a three-day duration (Fuzesi et al., 2007; Gelfo et al., 2012), adhering to dosage protocols as per previous research (Narvaez et al., 2015, 2016, 2018; Borroto-Escuela et al., 2021).

### Hippocampal cell proliferation assessment

Three weeks post-icv injections, the rats were deeply anesthetized with pentobarbital (Mebumal, 100 mg/kg, i.p.) and perfused transcardially with 4% PFA (paraformaldehyde, wt./vol, Sigma Aldrich, St. Louis, MI, USA). The brain tissues were precisely sectioned at 30 μm thickness across the dorsal hippocampus (posterior in primates) [from −1.60 to −5.30 Bregma; Paxinos and Watson, 2006 (Paxinos and Watson, 2006)]. The detection and examination of NPY have been established to be effective for up to 3 weeks after both central and peripheral application (Decressac et al., 2011; Corvino et al., 2014; Silveira Villarroel et al., 2018; Nahvi et al., 2021).

A series of antigen retrieval procedures were performed at 65°C for 90 min in saline sodium citrate buffer (pH 6; 10 nM sodium citrate). Subsequent treatments to eliminate endogenous peroxidases were carried out for 30 min in 0.6% H2O2. The sections were incubated overnight at 4°C with a primary antibody against PCNA (1:1500, P8825, Sigma, St. Louis, MO, USA), followed by a secondary antibody for 90 min (biotinylated anti-mouse IgG, 1/200, Vector Laboratories). Amplification was achieved with ExtrAvidin peroxidase (1:100, Sigma, St. Louis, MO, USA) in a dark room at room temperature for 1 h. Detection was realized with 0.05% diaminobenzidine (DAB; Sigma) and 0.03% H2O2 in PBS. Following multiple washes, sections were mounted, dehydrated, and cover-slipped. PCNA-labeled cells were analyzed via the optical fractionator method using unbiased stereological microscopy (Olympus BX51 Microscope, Olympus, Denmark), as previously detailed (Narvaez et al., 2018; Mirchandani-Duque et al., 2022; Alvarez-Contino et al., 2023) Supplementary Materials provide further specifications.

### Double immunolabeling technique

A double immunolabeling technique was employed to investigate the specific cell subpopulations involved in proliferation. The procedures were previously described and consist of an initial incubation with blocking and permeabilization solutions for 60 min each (Borroto-Escuela et al., 2021, 2022; Mirchandani-Duque et al., 2022). Pairs of primary antibodies mouse anti-PCNA (1:1500, P8825, Sigma, St. Louis, MO, USA)/rabbit anti-DCX (Abcam, ab18723, 1:2000) or mouse anti-PCNA (1:1500, P8825, Sigma, St. Louis, MO, USA)/rabbit anti-GFAP (Abcam, ab7260, 1:1500) were used to incubate the sections for 24 h at 4°C. Subsequently, incubations were performed with proper secondary antibodies: Donkey anti-mouse AlexaFluor 488 (Abcam, ab150105, 1:200) and Donkey anti-rabbit AlexaFluor 647 (Abcam, ab150075, 1:200). The sections were mounted with a fluorescent mounting medium containing DAPI (4’,6-diamidino-2-phenylindole) for nuclei detection (Abcam, ab104139). Quantitative analysis of the PCNA/DCX- and PCNA/GFAP-immunostained cells in the dentate gyrus of the dorsal hippocampus was conducted as described (Cohen et al., 2018; Mirchandani-Duque et al., 2022).

### Analysis of co-localization through *in situ* proximity ligation assay

To explore NPY1R-GALR2 co-localization in the dorsal dentate gyrus, the *in situ* proximity ligation assay (*in situ* PLA) method was utilized, specifically employing NaveniFlex Tissue GR Atto 647N, Navinci, Sweden. The procedure was performed on free-floating sections according to established protocols (Borroto-Escuela et al., 2021; Narváez et al., 2021). Brain slices were first conditioned with blocking buffer for 1 h at 37°C inside a pre-warmed humidity chamber, followed by overnight incubation at 4°C with the primary antibodies at optimal concentrations. Utilized antibodies included rabbit anti-GALR2 (1:100, Alomone Lab) and goat anti-NPYY1R (1:200, sc-21992, Santa Cruz Biotechnology INC, CA). Subsequently, the samples were rinsed three times, and a mixture of Navenibody goat and rabbit was applied for an hour at 37°C. Unbound probes were removed through washing, and Enzymes A and B were sequentially applied in a humidity chamber at 37°C for 60 and 30 min, respectively. After washing away excess connector oligonucleotides, the samples were treated with the rolling circle detection mixture (Enzyme C, Tex615) and incubated at 37°C for 90 min. The sections were then mounted and sealed with fluorescent mounting medium containing DAPI (Abcam, ab104139), stored at −20°C, and analyzed later through confocal microscopy as previously outlined (Narvaez et al., 2020; Borroto-Escuela et al., 2022; Diaz-Sanchez et al., 2023).

## Behavioral assessment

### Spatial memory evaluation in rats

Spatial hippocampal memory was evaluated using the object-in-place task based on spontaneous object exploration behaviors, drawing a contrast with the Morris water maze task (Harrison et al., 2009; Warburton and Brown, 2015).

A different cohort of animals were randomly allocated into five experimental groups: (1) aCSF: control group, (2) M1145-treated group (3 nmol), (3) NPY1R agonist-treated group receiving an NPYY1R agonist [Leu^31^,Pro^34^]NPY (3 nmol), (4) M1145 + NPY1R: group administered with both substances, and (5) M1145 + NPY1R + M871: group injected with M1145, [Leu^31^,Pro^34^]NPY, and the GALR2 antagonist (M871, 3 nmol) (*n* = 6 in each group).

Behavioral experiments were performed between 09:00 and 14:00 h. Animals were adapted to handling and were taken into the experimental room (80-90 lux) for at least 1 h to reach habituation and assigned randomly to the experimental groups. Rats were single-housed during the behavioral period. The task trials contain three phases: habituation, training, and test (Ampuero et al., 2013; Barker and Warburton, 2015) as follows:

Habituation: animals were handled for 2 days, then familiarized with the empty arena for 10 min (1 trial, 10 min), using a plastic open field, 100 × 100 × 60 cm (length × width × height).

Training: Every animal was placed in the middle of the arena 24 h after the habituation. The rats were allowed to explore four distinct objects which could not be displaced, during 3 min. The objects were placed in the corners of the arena 10 cm from the sidewall and were different in color and shape, with a similar weight and size. The objects were cleaned with 5% ethanol after the trial.

Test: Two objects were exchanged 24 h post-training and then the animals were re-exposed to explore the objects (1 trial, 3 min). The time spent sniffing or touching the object with the nose or forepaws was described as exploration. The time spent exploring the objects in the exchanged location (C) compared with the time spent exploring the objects in the same place (S) represented the discrimination ability. Then, the discrimination ratio was calculated as DI = (C − S)/(C + S). Intact object-in-place memory occurs when the animal spends more time examining the two objects in different locations than the same ones. Objects’ locations were counterbalanced between trials and between rats. Furthermore, the arena and the objects were carefully cleaned with 5% ethanol between sessions. The animals’ behavior was scored and analyzed blind to the treatment, using the Raton Time 1.0 software (Fixma S.L., Valencia, Spain). Moreover, the total exploration time and the locomotor activity between the animal groups were analyzed to validate that the treatments did not affect the exploration ability of the rats.

### Statistical analysis

Data were exhibited as mean ± SEM, with the number of samples denoted in the figure legends. GraphPad PRISM 8.0 (GraphPad Software, La Jolla, CA, USA) was harnessed for data examination. One-way ANOVA was conducted followed by the Newman-Keuls post-test for comparative analysis, or Student’s unpaired *t*-test where necessary. Significance thresholds were defined as *p < 0.05, **p < 0.01, ***p < 0.001.

## Results

### Enhancing effect of coadministration of GALR2 and the NPY1R agonists on the cell growth in the dorsal hippocampus

**FIGURE 1:**
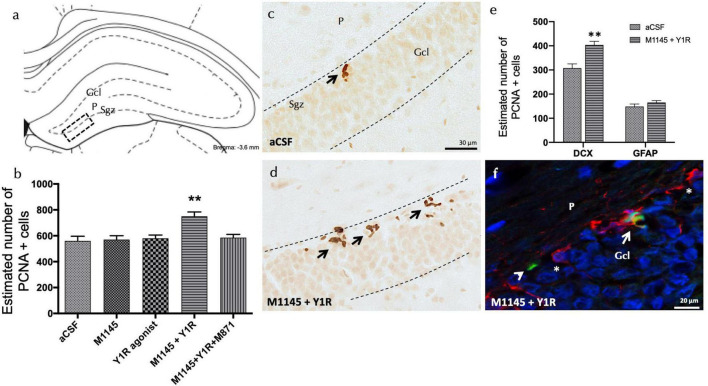
Coadministration of Galanin receptor 2 and NPY1R agonists increases cell proliferation in the dorsal dentate gyrus of adult rats. Proliferating cell nuclear antigen immunolabeling (PCNA +) in the dentate gyrus of the dorsal hippocampus, after the intracerebroventricular (icv) administration of Galanin 2 receptor agonist (M1145) and NPY1R receptor agonists, either alone or in combination with or without the GALR2 receptor antagonist (M871). **(A,D)** The majority of the PCNA-IR cells were located in the sub-granular zone (Sgz) of the dentate gyrus at the border between the granular cell layer (Gcl) and the polymorphic layer (P) of the dentate gyrus in the dorsal hippocampus. They appeared as groups of 3–4 cells (Bregma: –3.6 mm, according to the Paxinos and Watson stereotaxic atlas (Paxinos and Watson, 2006). **(B)** Quantification of total PCNA-IR cells in the dorsal hippocampal dentate gyrus. Data represent mean ± SEM, showing the differences between groups after the injections of aCSF, M1145, the NPY1R agonist [Leu31,Pro34]NPY, or the co-administration of both substances with or without M871. M1145 and the NPY1R agonist coadministration increased the number of cells with PCNA + expression in the dorsal hippocampus compared to the effects of the two peptides given alone and the control group. Furthermore, this effect was counteracted by the GALR2 antagonist M871. **p < 0.01 vs. the rest of the groups according to one-way ANOVA followed by Newman–Keuls *post-hoc* test. *n* = 4 in each group. M1145 and NPY1R agonist co-injection **(D)** increased the PCNA-IR cells in Sgz in the dentate gyrus compared with the control group **(C)**. Arrows indicate examples of clusters of PCNA + nerve cells. Dashed lines outline the Gcl of the dentate gyrus. **(E)** Quantification of PCNA-IR cells double-labeled with DCX or GFAP in either control or M1145 + NPY1R-administered rats revealed that NPY1R-GALR2 specifically acts onto the neuroblasts. Data represent mean ± SEM. **P < 0.01 vs. control according to Student’s unpaired *t*-test statistical analysis. **(F)** Representative photomicrograph illustrating DCX + /PCNA + cells (as indicated by white arrow), DCX-/PCNA + cells (as indicated by white arrowhead) and DCX + /PCNA- cells (as indicated by white asterisks) in the M1145 and NPY1R agonist group. aCSF, Control (artificial cerebrospinal fluid); M1145, Galanin receptor 2 agonist (3 nmol); NPY1R agonist, NPY1R receptor agonist [Leu31, Pro34]NPY (3 nmol); M1145 + NPY1R, co-administration of M1145 and NPY1R agonist; M1145 + NPY1R + M871, co-administration of M1145, NPY1R, and GALR2 antagonist M871 (3 nmol).

Through employing the proliferating cell nuclear antigen (PCNA), the joint influence of the GALR2 agonist M1145 and the NPY1R agonist on the adult dorsal hippocampal cell growth was explored. The specific area of interest was the sub-granular zone (Sgz) of the dentate gyrus. The results exhibited a notable augmentation in cell proliferation with the combined administration of icv M1145 and icv NPY1R agonist, as indicated by the quantity of PCNA-IR cells (one-way ANOVA, F4,15 = 6.72, *p* < 0.01, Newman–Keuls *post-hoc* test: *p* < 0.01) (Figures 1A, B, D), including M1145 (Newman–Keuls *post-hoc* test: *p* < 0.01) and NPY1R agonist groups (Newman–Keuls *post-hoc* test: *p* < 0.01) (Figures 1A, B, D). The concurrent addition of GALR2 antagonist M871 negated the impact of M1145 and the NPY1R agonist within the dentate gyrus (Newman–Keuls *post-hoc* test: *p* < 0.01) (Figure 1B), thereby confirming the role of GALR2 in stimulating cell growth in the dorsal hippocampus via the NPY1R/GALR2 agonist interaction.

However, icv administration of the NPY1R agonist alone showed no significant alteration in the count of PCNA-IR cells within the Sgz of the dentate gyrus (Figures 1A, B), similar to the administration of M1145 alone, in contrast to the control group (Figures 1A–C).

### Identification of affected cellular types through the icv injection of M1145 and the NPY1R agonist

The focus then shifted to identifying the cell types influenced by icv M1145 and NPY1R agonist administration. Quantification of PCNA labeled cells was performed to recognize the co-expression of either doublecortin (DCX)-expressing neuroblasts or Glial Fibrillary Acidic Protein (GFAP)-expressing dormant radial stem cells (Figure 1E). The number of PCNA + /DCX + cells saw a significant increase after the icv M1145 and NPY1R agonist administration as compared to the control (*t* = 4.114, df = 6; *p* < 0.01)(Figure 1F). However, no considerable change was detected in the count of PCNA + /GFAP + cells within M1145-NPY1R-treated subjects (*t* = 1.254, df = 6; *p* < 0.26). This outcome signified that the combination of M1145 and the NPY1R agonist promotes neuroblast (DCX +) proliferation, with no apparent influence on inactive radial stem (GFAP +).

### Coactivation of GALR2 and NPY1R enhances PLA positive signals

To explore the cellular mechanisms related to the observed cell proliferation effects, an *in situ* proximity ligation assay (PLA) was performed in the dorsal hippocampal dentate gyrus (DG). The study focused on the GALR2-NPY1R co-localization following icv administration of M1145 and NPY1R agonists.

**FIGURE 2:**
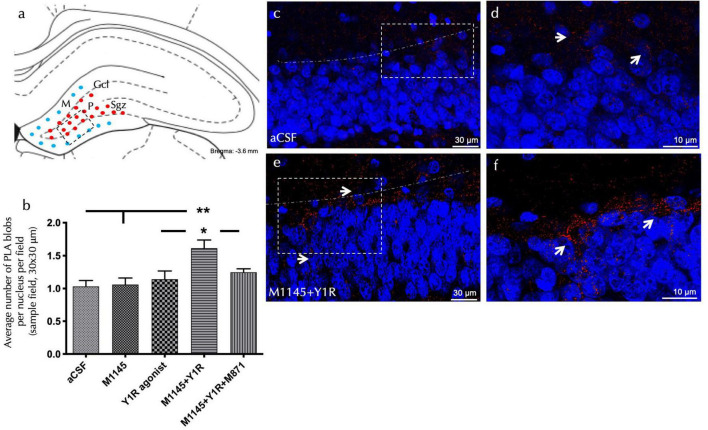
Detection of GALR2/NPYNPY1R co-localization by *in situ* PLA in the dorsal hippocampal dentate gyrus (DG). The *in situ* PLA Technology enables visualization of protein-protein interaction using one primary antibody for each target protein **(A)** The diagram illustrated the presence of positive PLA signals (red circles) mainly in the subgranular zone (Sgz) of the dentate gyrus at the border between the granular cell layer (Gcl) and polymorphic layer (P) of the dentate gyrus in the ventral hippocampus. PLA positive signals were also observed in the polymorphic layer. Blue filled circles indicate negative PLA signal in the molecular layer (M). (Bregma: –3.6 mm; according to the Paxinos and Watson (2006) stereotaxic atlas (Paxinos and Watson, 2006). **(B)** Quantification of PLA signals in Sgz was performed by measuring red PLA positive blobs per nucleus per sampled field by an experimenter blind to treatment conditions. This effect was blocked by treatment with the GALR2 antagonist M871. *P < 0.05 vs. NPY1R agonist and M1145 + NPY1R + M871 groups; **P < 0.01 vs. Control and M1145 groups according to one-way ANOVA followed by Newman–Keuls *post-hoc* test (*n* = 4 in each group). Data are expressed as mean ± SEM. **(C–F)** Representative microphotographs of the significant increase in the density of Y1RGALR2 heteroreceptor complexes (PLA clusters) after M1145 and Y1R agonists treatment **(C,D)** compared with the control group **(E,F)**. Receptor complexes are shown as red PLA blobs (clusters, indicated by white arrows) found in high densities per cell in a large number of nerve cells using confocal laser microscopy. Dashed lines outline the Gcl of the dentate gyrus. The nuclei are shown in blue by DAPI staining. aCSF, Control (artificial cerebrospinal fluid); M1145, Galanin receptor 2 agonist (3 nmol); NPY1R agonist, NPY1R receptor agonist [Leu31, Pro34]NPY (3 nmol); M1145 + NPY1R, co-administration of M1145 and NPY1R agonist; M1145 + NPY1R + M871, co-administration of M1145, NPY1R, and GALR2 antagonist M871 (3 nmol).

Explicit PLA-positive red puncta were observed in the sub granular zone and the polymorphic layer of the dorsal DG (Figure 2A). Quantification of PLA puncta density authenticated an increased density of these puncta after M1145 and YR1 agonist co-administration, when contrasted to the control (one-way ANOVA, F4, 15 = 5.3, *p* < 0.01, Newman-Keuls *post-hoc* test: *p* < 0.01); M1145 group (Newman-Keuls *post-hoc* test: *p* < 0.01), or NPY1R agonist solely (Newman-Keuls *post-hoc* test: *p* < 0.05) (Figures 2B–F). In alignment with the PCNA-IR profile, no significant increase was seen with the icv NPY1R agonist alone in the density of PLA-positive red puncta (Figure 2B). The M1145 on its own showed no impact on the puncta when compared to the control (Figure 2B). However, the GALR2 antagonist M871 entirely negated this increase (Newman-Keuls *post-hoc* test: *p* < 0.05) (Figure 2B), underscoring the role of GALR2 in this interaction. The specificity of the signal was affirmed through the lack of PLA puncta in the molecular layer of the DG, an area believed to be devoid of GALR2 (O’Donnell et al., 1999).

### Improved spatial memory performance following GALR2 agonist and NPY1R agonist coadministration, microinjected ICV

**FIGURE 3:**
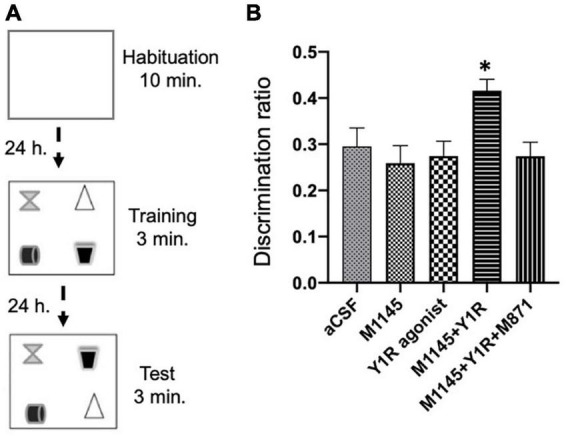
Spatial memory assessment after M1145 and the NPY1R agonist alone and combined in the object-in-place memory task. **(A)** Schematic representation of the trials completed in the object-in-place task. The animals performed the task in three phases, divided 24 h from each other, where they explored freely for 10 min in the habituation phase without objects, 3 min in the training phase with four different objects, and finally, 3 min in the test phase with two of the objects with the exchanged position. To achieve memory consolidation, the pharmacological treatments were administered intracerebroventricularly (icv) to the different groups of animals 3 weeks before the testing phase. **(B)** Performance on the object-in-place task showing the ability of rats to discriminate the exchanged objects 3 weeks after the icv administration of M1145 in combination with the NPY1R agonist. An improvement in the object-in-place performance was observed after M1145 and NPY1R agonist co-administration, and this effect is counteracted by the GAL 2 receptor (GALR2) antagonist M871. Data are presented as the mean ± SEM of the discrimination ratio on the test phase. *n* = 6 animals in each group. *p < 0.05 vs. the rest of the groups according to one-way ANOVA followed by Newman–Keuls *post-hoc* test. aCSF, Control (artificial cerebrospinal fluid); M1145, Galanin receptor 2 agonist (3 nmol); NPY1R agonist, NPY1R receptor agonist [Leu31, Pro34]NPY (3 nmol); M1145 + NPY1R, co-administration of M1145 and NPY1R agonist; M1145 + NPY1R + M871, co-administration of M1145, NPY1R, and GALR2 antagonist M871 (3 nmol).

We executed the object-in-place task 3 weeks after the icv injections. During a 10-minute habituation phase, rats roamed freely without objects, followed by a 3-minute training phase with four diverse objects. The test phase lasted 3 min, involving two objects with swapped positions to gauge the effects of the drugs on the object-in-place memory performance (Figure 3A).

The icv coadministration of GALR2 agonist M1145 and NPY1R after the acquisition phase notably enhanced object-in-place memory retention after a 24-hour interval compared to other groups (one-way ANOVA, F4, 25 = 3.69, *p* < 0.05; Newman–Keuls *post-hoc* test: *p* < 0.05; Figure 3B). The enhancement of memory performance was attributed to GALR2, as the inclusion of the GALR2 antagonist M871 offset the boost in memory (Newman–Keuls *post-hoc* test: *p* < 0.05; Figure 3B) instigated by the simultaneous administration of GALR2 and NPY1R agonists. Neither M1145 nor the NPY1R agonist independently had any effect on the object-in-place memory task (Figure 3B) relative to the control.

Furthermore, we scrutinized the total exploration time throughout the training and testing sessions and found that neither the exploration ability nor the innate motor behavior of the subjects were influenced by the treatments. Significantly, the subjects displayed a marked preference for the translocated objects versus the ones that stayed in place, as revealed by within-group analyses: Control (*t* = 9.26; df = 5; *p* < 0.001), M1145 (*t* = 6.80; df = 5; *p* < 0.01), NPY1R agonist (*t* = 8.90; df = 5; *p* < 0.001), M1145 + NPY1R (*t* = 19.77; df = 5; *p* < 0.001), and M1145 + NPY1R + M871 (*t* = 7.89; df = 5; *p* < 0.001). In addition, the time spent exploring the rearranged objects was significantly elevated in the M1145 + NPY1R group relative to control (one-way ANOVA, F4, 25 = 10.15, *p* < 0.001; Newman–Keuls *post-hoc* test: *p* < 0.001), M1145 (Newman–Keuls *post-hoc* test: *p* < 0.001), NPY1R agonist (Newman–Keuls *post-hoc* test: *p* < 0.01), and M1145 + NPY1R + M871 (Newman–Keuls *post-hoc* test: *p* < 0.01) groups.

## Discussion

Evidence is presented demonstrating Indications of a sustained increase in cell multiplication within the dorsal dentate gyrus (DG) of the hippocampus after combined icv administration of GALR2 and NPY1R agonists. Consistent with these findings, our observations reveal an absence of effect by the NPY1R agonist alone on cellular multiplication in the dorsal DG (Borroto-Escuela et al., 2022; Mirchandani-Duque et al., 2022).This aligns with earlier results that found no variance in dorsal hippocampus DG cell growth following NPY injection under physiological conditions in rodent (Corvino et al., 2014). In relation to the application of the GALR2 agonist on its own, no effects were detected on dorsal hippocampal cell multiplication. The present findings indicated that icv treatment with the GALR2 agonist M1145 combined with the NPY1R agonist significantly enhanced the number of cells with proliferating cell nuclear antigen (PCNA), visualized with immunoreactivity in the sub-granular zone of the dentate gyrus. The number of doublecortin (DCX) immunoreactive cells, being a microtubule-associated protein, was also found to be significantly increased in the double labeled experiments with PCNA. This was not true for glial fibrillary acidic protein (GFAP) immunoreactivity upon double labeling with PCNA. No change was observed in the number of GFAP immunoreactive cells including astrocytes. Thus, Indications of a sustained increase in cell multiplication within the dorsal dentate gyrus (DG) of the hippocampus were obtained after combined icv administration of GALR2 and NPY1R agonists. Additionally, our findings illustrate how M145 and NPY1R agonist coadministration prompted short-term neuroblast growth in the ventral hippocampal DG (Alvarez-Contino et al., 2023). Previous observations reveal an absence of effect by the NPY1R agonist alone on cellular multiplication in the dorsal DG (Deng et al., 2010; Borroto-Escuela et al., 2018, 2022; Mirchandani-Duque et al., 2022). This aligns with earlier results that found no variance in dorsal hippocampus DG cell growth following NPY injection under physiological conditions in rats (Corvino et al., 2014). Interestingly, our observation contrasts with studies conducted on mice, which indicated that exogenous NPY promotes DG cell proliferation in the dorsal hippocampus under similar conditions (Howell et al., 2007; Decressac et al., 2011). This discrepancy underscores potential species-specific differences in neurogenesis between rats and mice, as suggested in other studies (Ray and Gage, 2006; Radic et al., 2017). Notably, research indicates that hippocampal neurons born in adulthood appear to be more abundant, mature at a quicker rate, and have a more pronounced role in behavioral processes in rats compared to mice (Snyder et al., 2009). It is important to note that previous reports demonstrating the proliferative effects of NPY on rat neural stem cells were predominantly based on *in vitro* systems (Howell et al., 2003, 2005). These conditions exhibit significant differences from our *in vivo* experimental approach, possibly accounting for the variance in outcomes. Furthermore, considering the context of pathological conditions where neurogenesis is affected, the role of the NPY1R agonist might differ. For instance, NPY was found to be effective in increasing cell proliferation in the dorsal DG in a hippocampal model of neurodegeneration (Corvino et al., 2014). This suggests a potential therapeutic relevance of NPY1R agonist in such conditions. It is particularly noteworthy that the NPY1R agonist has shown promising results in rescuing impaired learning and memory in a rat model of Alzheimer’s disease (Rangani et al., 2012) However, it’s important to highlight that, despite these promising results in pathological models, there is currently limited evidence to suggest a similar impact of NPY1R agonists on learning and memory under physiological conditions. This gap in knowledge emphasizes the need for further research to delineate the specific contexts in which NPY1R agonists can be most effectively utilized, especially in contrasting pathological and physiological conditions.

In relation to the application of the GALR2 agonist on its own, no effects were detected on dorsal hippocampal cell multiplication, underlining the potential importance of the putative NPY1R-GalR heterocomplexes in cognition. It is in line with the hypothesis that various heteroreceptor complexes formed in the brain, especially in the tel-and diencephalon give the molecular mechanism for learning and memory (Fuxe et al., 2014; Borroto-Escuela et al., 2015, 2018).

To understand the cellular effects following coadministration of NPY1R and GALR2 agonists we studied the presence of putative NPY1R-GALR2 heteroreceptor complexes on dorsal DG. As *in situ* PLA typically provides a resolution in the range of 25−30 nm when secondary IgG antibodies are used, we cannot exclude the possibility that in our current experiments, we may detect NPY1R-GALR2 heteroreceptor complexes and/or the co-location of NPY1R and GALR2 within the same cell membrane nanodomains (hereafter referred to as heterocomplexes) (Hernández-Mondragón et al., 2023). This technique permits accurate location of putative NPY1R-GALR2 heteroreceptor complexes. A noticeable increase in putative heteroreceptor complexes was found after 3 weeks of sub-chronic stimulation of both receptor protomers in the dorsal DG. The GALR2 involvement was demonstrated by the blocking effect of GALR2 antagonist M871 on the formation of the NPY1R-GalR2 heterocomplexes. Additionally, the absence of NPY1R-GALR2 heteroreceptor complexes in the molecular layer of the dentate gyrus aligns with the unavailability of GALR2 in this area (O’Donnell et al., 1999). While our study demonstrates co-localization of these receptors in the hippocampal dentate gyrus, this alone does not definitively confirm the formation of GALR2-NPY1R heteroreceptor complexes. Previous research in various brain regions, including the amygdala, hippocampus, hypothalamus, and prefrontal cortex, has suggested functional interactions between these receptors that could be attributed to the formation of such complexes (Narvaez et al., 2015, 2016, 2018; Borroto-Escuela et al., 2021, 2022; Mirchandani-Duque et al., 2022; Diaz-Sanchez et al., 2023). These studies have indicated potential involvement in processes such as binding, internalization, intracellular signaling and synergistic or antagonistic effects following dual receptor activation or inhibition which are indicative of a more complex interplay beyond mere physical proximity. However, to unequivocally demonstrate the structural formation and functional interdependency of GALR2-NPY1R heteroreceptor complexes, further sophisticated experimental approaches are warranted. Techniques such as Co-Immunoprecipitation (CoIP), Fluorescence Resonance Energy Transfer (FRET), and Bioluminescence Resonance Energy Transfer (BRET) could provide more direct evidence of these complexes. Additionally, employing advanced imaging techniques like super-resolution microscopy could offer a deeper insight into the nanoscopic interactions between these receptors.

In light of these considerations, our current study provides an understanding of the possible interactions between GALR2 and NPY1R, paving the way for more detailed investigations into the existence of these heteroreceptor complexes. Such research would not only clarify the mechanistic aspects of GALR2-NPY1R interactions but also potentially reveal novel therapeutic targets for neuropsychiatric disorders.

Our present findings may point to elevated intracellular signaling in the dorsal DG neuroblasts associated with the expansion of putative NPY1R-GALR2 heteroreceptor complexes and their potential link to the observed functional actions. Our behavioral results, revealing a substantial boost in object-in-place memory consolidation 3 weeks post-treatment, are in harmony with earlier research highlighting the cognitive-boosting abilities of GALR2 and NPY1R activation within 24 h (Borroto-Escuela et al., 2022; Mirchandani-Duque et al., 2022). The collaborative interaction between putative GalR2-NPY1R heterocomplexes suggest its participation in memory consolidation. It becomes especially pertinent when the individual administration of M1145 or the NPY1R agonist is observed not to impact memory performance significantly. The anticipated forward effects of neurogenesis on memory indicates that the addition of new neurons can play a critical role prior to learning novel contexts. For instance, inhibiting immature adult-born neurons in mice resulted in an inability to form durable spatial memories and adopt precise spatial learning strategies (Deng et al., 2009; Garthe et al., 2009). Furthermore, neurogenesis appears necessary for certain hippocampal-dependent tasks and is heightened when neurogenesis is upregulated (Deng et al., 2010; Sahay et al., 2011). Intriguingly, only the DG among hippocampal subregions showed a decrease in NPY-IR fibers in an AD rat model (Rangani et al., 2012). Supporting our findings, genetic enhancement of neurogenesis in the dorsal DG of the hippocampus led to improved spatial learning in aged rodents (Berdugo-Vega et al., 2020). Additionally, evidence has revealed that proliferating dentate granule cells are integrated into the hippocampal neuronal network as early as 2−3 weeks post-birth in rats (Snyder et al., 2009; Gu et al., 2012; Nakashiba et al., 2012). Using pathological animal models for disturbances in learning and memory would reinforce the current observations. Thus, additional investigation into NPY and GAL receptor interactions in the dorsal hippocampus in pathological models of cognition disturbances are warranted.

Our results may foster clinical advancements in the creation of novel heterobivalent or multitargeting drugs that function as agonist pharmacophores on NPY1R–GALR2 heterocomplexes in the dorsal hippocampus. Future therapeutic strategies might center on these heterocomplexes for manipulating neurogenic hippocampal activity in learning and memory. Additionally, evaluating the dynamics of heteroreceptor complexes could serve as a fresh biomarker to pinpoint drug targeting with local precision. In light of our findings on the NPY1R-GALR2 interaction and the formation of potential heteroreceptor complexes, it is pertinent to discuss the implications of these results for the development of novel therapeutic interventions targeting learning and memory disorders. The intricate role of these receptor interactions in cognitive processes suggests that modulating the NPY1R-GALR2 heterocomplex could be a promising strategy for treating various neurocognitive disorders. Several learning and memory disorders, such as Alzheimer’s disease, age-related cognitive decline, and certain forms of amnesia, could potentially benefit from therapies targeting the NPY1R-GALR2 pathway. For example, Alzheimer’s disease, characterized by progressive memory loss and cognitive decline, has been linked to dysregulation in neuropeptide systems, including NPY (Flood et al., 1987; Li et al., 2019). Modulating the NPY1R-GALR2 complex in Alzheimer’s patients could potentially mitigate some of the cognitive deficits associated with the disease. Similarly, age-related cognitive decline, which often involves impairments in spatial memory and learning, might be alleviated through interventions targeting these heteroreceptor complexes. Studies have shown that NPY and its receptors play a significant role in neurogenesis and synaptic plasticity, processes that are crucial for learning and memory (Duarte-Neves et al., 2016; Gotzsche and Woldbye, 2016). In addition, certain forms of amnesia, particularly those linked to hippocampal dysfunction, could be addressed through this pathway.

In conclusion, the NPY1R-GALR2 interaction presents a novel avenue for therapeutic intervention in various learning and memory disorders. Further research is necessary to fully understand the therapeutic potential of modulating this heteroreceptor complex and to develop targeted treatments that can effectively address the specific pathological mechanisms of these disorders. The ensuing findings justify the planning of subsequent clinical trials involving the administration of NPY1R and GALR2 agonists. There exist huge numbers of various types of heteroreceptor complexes in the brain networks, including the hippocampus. The hypothesis has been introduced that they represent the molecular basis for learning and memory in the brain (Fuxe et al., 2014; Borroto-Escuela et al., 2015, 2018).

## Data availability statement

The original contributions presented in this study are included in this article/Supplementary materials, further inquiries can be directed to the corresponding author.

## Ethics statement

The animal study was approved by the University of Málaga’s Local Animal Ethics, Care, and Use Committee, Spain (CEUMA 45-2022-A). The study was conducted in accordance with the local legislation and institutional requirements.

## Author contributions

RB-C: Data curation, Methodology, Writing – original draft. AH-G: Formal Analysis, Investigation, Software, Writing – original draft. PA: Data curation, Methodology, Writing – review & editing. EB-R: Formal Analysis, Investigation, Software, Writing – original draft. PS-C: Funding acquisition, Investigation, Resources, Supervision, Writing – review & editing. NG-C: Funding acquisition, Investigation, Resources, Supervision, Validation, Writing – review & editing. KF: Conceptualization, Funding acquisition, Resources, Supervision, Validation, Visualization, Writing – original draft, Writing – review & editing. DB-E: Conceptualization, Funding acquisition, Investigation, Resources, Supervision, Validation, Visualization, Writing – original draft, Writing – review & editing. MN: Conceptualization, Funding acquisition, Investigation, Project administration, Resources, Supervision, Visualization, Writing – original draft, Writing – review & editing.
