# Supplementary material for: Neuropeptide Y receptor 1 and galanin receptor 2 (NPY1R-GALR2) interactions in the dentate gyrus and their relevance for neurogenesis and cognition
Source: Front Cell Neurosci. 2024 Feb 14;18:1323986. doi: 10.3389/fncel.2024.1323986 (PMC10902914; doi:10.3389/fncel.2024.1323986)
Supplement: Supplementary file 1 [file Data_Sheet_1.DOCX]

***Supplementary Material***

**Y1R-GALR2 interactions in the dentate gyrus and their relevance for neurogenesis and cognition**

Rasiel Beltrán-casanueva 1,2**;** Aracelis Hernández-García 1,2**;** Paula de Amo García 3**;** Encarnación Blanco-Reina 3; Pedro Serrano-Castro 3,4,5**;** Natalia García-Casares 3**;** Kjell Fuxe 1***;** Dasiel Borroto-Escuela 1,2*; Manuel Narváez 3,4*.

1 Department of Neuroscience, Karolinska Institutet, Stockholm, Sweden. Email: (KF) [Kjell.Fuxe@ki.se](mailto:Kjell.Fuxe@ki.se); (DOBE) [Dasiel.Borroto-Escuela@ki.se](mailto:Dasiel.Borroto-Escuela@ki.se); (RBC) [rasiel.beltran@stud.ki.se](mailto:rasiel.beltran@stud.ki.se).

2 Receptomics and Brain disorders lab, Edificio Lopez-Peñalver, Instituto de Investigación Biomédica de Málaga, Facultad de Medicina, Universidad de Málaga, Spain. E-mail: (DOBE) [*dasiel@uma.es*](mailto:dasiel@uma.es)

3 Instituto de Investigación Biomédica de Málaga, Facultad de Medicina, Universidad de Málaga., Málaga, Spain, [eblanco@uma.es](mailto:eblanco@uma.es); [Pedro.serrano.c@gmail.com](mailto:Pedro.serrano.c@gmail.com) ; [Nagcasares@uma.es](mailto:Nagcasares@uma.es) ; [mnarvaez@uma.es](mailto:mnarvaez@uma.es)

4 Vithas Málaga, Vithas Málaga. Grupo Hospitalario Vithas, Málaga, Spain,

5 Instituto de Investigación Biomédica de Málaga, Unit of Neurology, Hospital Regional Universitario de Málaga, Málaga, Spain

Correspondence: mnarvaez@uma.es (MN)

**Intracerebral cannulations**

Rats anesthetized intraperitoneally with Equitesin (3,3ml/Kg; chloral hydrate 2.1 g, sodium pentobarbital 0.46 g, MgSO4 1.06 g, propylene glycol 21.4 ml, ethanol (90%) 5.7 ml, H_2_O 3 ml; Sigma Aldrich) were implanted with a chronic 22- gauge stainless-steel guide cannula (Plastics One In) into the right lateral cerebral ventricle using the following stereotaxic coordinates: +1.4mm lateral, -1mm posterior to the bregma, and 3.6mm below the surface of the skull (Paxinos and Watson, 2006). After surgery, animals were individually housed and allowed recovery for 7 days. This method of anesthesia, cannulation and postsurgical care has been previously standarized (Narváez et al., 2015; Narváez et al., 2016; Narváez et al., 2018; Borroto-Escuela et al., 2021; Mirchandani-duque et al., 2022).

**Intracerebroventricular administration of peptides**

Cannulated rats were randomly allocated to different groups. Peptides were freshly prepared, dissolved in aCSF and injected into the right lateral ventricle. The total volume was 5 μl per injection with an infusion time of 1 min. The Y1R agonist [Leu31,Pro34]NPY, GALR2 agonist M1145 and GALR2 antagonist M871 (Ki=13.1 and 420 nM for GALR2 and GALR1 respectively) were obtained from Tocris Bioscience (Bristol, UK). Experimental groups and the n size of each group is indicated in the different procedures. After the experiments, brains were removed, testing the placement of the cannula for icv injection by cutting the brain in the coronal plane in a Cryostat (HM550, Microm International). These procedures of intracerebroventricular (icv) injections and preparation of artificial cerebrospinal fluid (aCSF) have already been standarized in our laboratory (Narváez et al., 2015; Narváez et al., 2016; Narváez et al., 2018; Borroto-Escuela et al., 2021; Mirchandani-duque et al., 2022).

**Counting Procedure**

PCNA-labeled cells were counted with an Olympus BX51 microscope, Olympus, Denmark interfaced with a computer and a colour JVC digital video camera. For stereological analysis, sampling of positive cells was performed throughout the dentate gyrus of the dorsal hippocampus in the rostrocaudal dimension using the optical fractionator, according to Paxinos & Watson atlas coordinates (Paxinos & Watson, 2006). This method combines the optical dissector with a fractionator sampling scheme to exclude volume divergences (Gundersen et al., 1988). Counterstaining with phase contrast allowed delineation of different areas in each section (Paxinos and Watson, 2006). Numbers of positive cells were quantified in at least five representative 150 μm, evenly spaced sections per animal (4 rats per group). A random set of sampling frames with a known area (α frame) was generated for each section using the C.A.S.T. Grid (Olympus; Albertslund, Denmark). After the objects were counted (ΣQ-) the total number of positive cells were estimated as: N = ΣQ- x fs x fa x fh (Gundersen et al., 1988), where fs is the numerical fraction of the section used, fa is the areal fraction and fh is the linear fraction of section thickness. The coefficient of error (CE) for each estimation and animal ranged from 0.05 to 0.1. The total CE of each group ranged from 0.07 to 0.08. Counting of labelled cells was set starting at 5 μm below the surface and focusing through the 20 μm section optical plane, and the number of counting frames used was 90-110 per animal. We have used this stereological procedure is previous studies (Narvaez et al., 2016; Narvaez et al., 2018; Borroto-escuela et al., 2022; Mirchandani-duque et al., 2022).

**Y1R-GALR2 Double immunofluorescence**

An initial incubation with blocking (5% goat serum) and permeabilization (0.3% triton X100 in PBS) solutions were done during 60 min each. Pairs of primary antibodies rabbit anti-GALR2 (Alomone Lab, 1:100) / goat anti-NPYY1R (sc-21992 Santa Cruz Biotecnology INC, EEUU, 1:100) were used to incubate the sections for 24 hours at 4 °C. Subsequently, incubations were performed with proper secondary antibodies: mouse anti- goat DyLight 488 (Jackson Laboratories InmunoResearch, 1:100) and mouse anti-rabbit DyLight 549 (Jackson inmunoResearch Laboratories, 1:100). The sections were mounted with a fluorescent mounting medium containing DAPI (4’,6-diamidino-2-phenylindole) for nuclei detection (Abcam, ab104139).

**
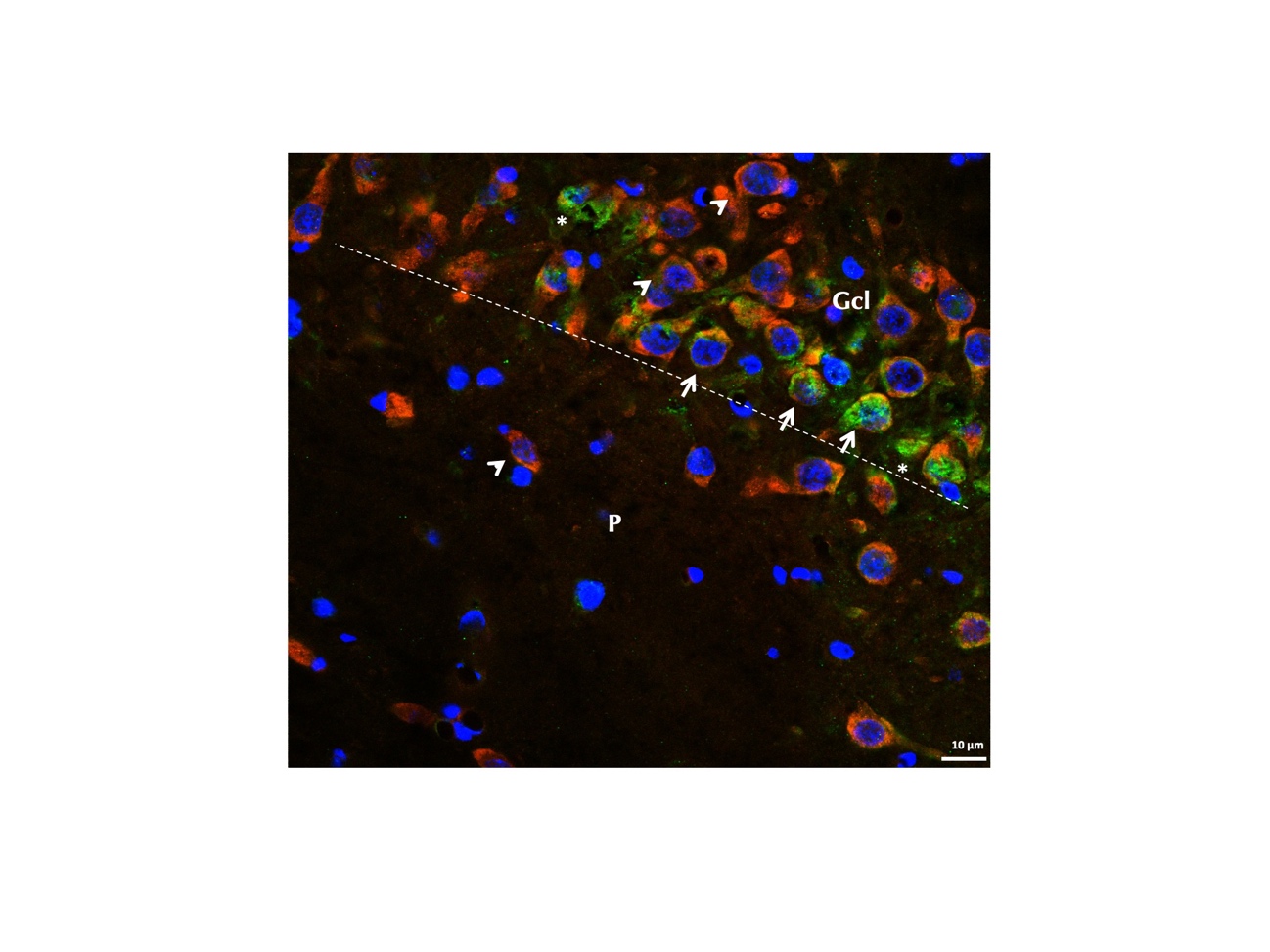
Supplementary figure 1.** Representative laser scanning confocal micrographs illustrating the polymorphic (P), granular (Gcl) layers of the dentate gyrus (Bregma: -3.6 mm). Nuclei are shown in blue (DAPI). Representative laser scanning confocal micrograph showing the colocalization of endogenous Neuropeptide Y Y1 receptor (Y1R) and Galanin receptor subtype 2 (GALR2) (Yellow-orange, as indicated by white arrows) in a subpopulation of neurons in the subgranular zone of the dentate gyrus. Y1R positive cells (green) are indicated by white arrowheads and GALR2-IR cells (red) are indicated by white asterisks.

**References**

Borroto-Escuela, D. O., Fores, R., Pita, M., Barbancho, M. A., Zamorano-Gonzalez, P., Casares, N. G., . . . Narvaez, M. (2022). Intranasal Delivery of Galanin 2 and Neuropeptide Y1 Agonists Enhanced Spatial Memory Performance and Neuronal Precursor Cells Proliferation in the Dorsal Hippocampus in Rats. Front Pharmacol, 13, 820210. doi:10.3389/fphar.2022.820210

Gundersen, H.J., Bagger, P., Bendtsen, T.F., Evans, S.M., Korbo, L., Marcussen, N., Moller, A., Nielsen, K., Nyengaard, J.R., Pakkenberg, B., and Et Al. (1988). The new stereological tools: disector, fractionator, nucleator and point sampled intercepts and their use in pathological research and diagnosis. APMIS 96, 857-881.

Mirchandani-Duque, M., Barbancho, M. A., Lopez-Salas, A., Alvarez-Contino, J. E., Garcia-Casares, N., Fuxe, K., . . . Narvaez, M. (2022). Galanin and Neuropeptide Y Interaction Enhances Proliferation of Granule Precursor Cells and Expression of Neuroprotective Factors in the Rat Hippocampus with Consequent Augmented Spatial Memory. Biomedicines, 10(6). doi:10.3390/biomedicines10061297

Narvaez, M., et al. (2015). "Galanin receptor 2-neuropeptide Y Y1 receptor interactions in the amygdala lead to increased anxiolytic actions." Brain Struct Funct 220(4): 2289-2301.

Narvaez, M., et al. (2016). "Galanin receptor 2-neuropeptide Y Y1 receptor interactions in the dentate gyrus are related with antidepressant-like effects." Brain Struct Funct 221(8): 4129-4139

Narvaez, M., et al. (2018). "A Novel Integrative Mechanism in Anxiolytic Behavior Induced by Galanin 2/Neuropeptide Y Y1 Receptor Interactions on Medial Paracapsular Intercalated Amygdala in Rats." Front Cell Neurosci 12: 119

Paxinos, G., and Watson, C. (2006). *The rat brain in stereotaxic coordinates: hard cover edition.* Elsevier.
